# Left ventricular shape variation in asymptomatic populations: the multi-ethnic study of atherosclerosis

**DOI:** 10.1186/s12968-014-0056-2

**Published:** 2014-07-30

**Authors:** Pau Medrano-Gracia, Brett R Cowan, Bharath Ambale-Venkatesh, David A Bluemke, John Eng, John Paul Finn, Carissa G Fonseca, Joao AC Lima, Avan Suinesiaputra, Alistair A Young

**Affiliations:** 1 Department of Anatomy with Radiology, University of Auckland, Auckland, New Zealand; 2 The Donald W. Reynolds Cardiovascular Clinical Research Center, The Johns Hopkins University, Baltimore, USA; 3 National Institute of Biomedical Imaging and Bioengineering, Bethesda, Maryland, USA; 4 Department of Radiology, University of California, Los Angeles (UCLA), Los Angeles, USA

**Keywords:** Cardiovascular magnetic resonance, Atlas, Principal component analysis

## Abstract

**Background:**

Although left ventricular cardiac geometric indices such as size and sphericity characterize adverse remodeling and have prognostic value in symptomatic patients, little is known of shape distributions in subclinical populations. We sought to quantify shape variation across a large number of asymptomatic volunteers, and examine differences among sub-cohorts.

**Methods:**

An atlas was constructed comprising 1,991 cardiovascular magnetic resonance (CMR) cases contributed from the Multi-Ethnic Study of Atherosclerosis baseline examination. A mathematical model describing regional wall motion and shape was used to establish a coordinate map registered to the cardiac anatomy. The model was automatically customized to left ventricular contours and anatomical landmarks, corrected for breath-hold mis-registration between image slices. Mathematical techniques were used to characterize global shape distributions, after removal of translations, rotations, and scale due to height. Differences were quantified among ethnicity, sex, smoking, hypertension and diabetes sub-cohorts.

**Results:**

The atlas construction process yielded accurate representations of global shape (errors between manual and automatic surface points in 244 validation cases were less than the image pixel size). After correction for height, the dominant shape component was associated with heart size, explaining 32% of the total shape variance at end-diastole and 29% at end-systole. After size, the second dominant shape component was sphericity at end-diastole (13%), and concentricity at end-systole (10%). The resulting shape components distinguished differences due to ethnicity and risk factors with greater statistical power than traditional mass and volume indices.

**Conclusions:**

We have quantified the dominant components of global shape variation in the adult asymptomatic population. The data and results are available at cardiacatlas.org. Shape distributions were principally explained by size, sphericity and concentricity, which are known correlates of adverse outcomes. Atlas-based global shape analysis provides a powerful method for quantifying left ventricular shape differences in asymptomatic populations.

**Trial registration:**

ClinicalTrials.gov NCT00005487

## Background

Adverse remodeling of the left ventricle (LV) can be defined as a change in shape due to cardiovascular disease which is associated with worse prognostic outcome [1]. For example, increased end-systolic volume is a predictor of mortality in patients after myocardial infarction [2]. LV sphericity, or length to width ratio, has also been associated with decreased survival [3] and is predictive of subsequent volume increase [4] in these patients. Concentric hypertrophy, or wall to cavity ratio, is predictive of adverse events in hypertension [5]. However, characterization of global shape change in health and disease has been difficult due to the lack of a standardized map of the heart. In order to quantify the large-scale shape changes undergone in patients with clinical disease, the variation present in the asymptomatic population must first be characterized.

In the Framingham study of asymptomatic individuals, LV chamber dimension was associated with increased adverse events [6], as were lower systolic dimension change [7], and hypertrophy [8]. However, simple volume or dimension measures fail to capture the wealth of data available in modern non-invasive imaging examinations. Access to this information would be very useful for the quantification of subclinical disease in the asymptomatic population and the evaluation of disease severity in patient groups.

The Multi-Ethnic Study of Atherosclerosis (MESA) was initiated in July 2000 to investigate the prevalence, correlates, and progression of subclinical cardiovascular disease in a population-based sample of 6,814 men and women aged 45–84 years [9]. Cardiovascular magnetic resonance (CMR) was used in MESA to evaluate cardiac mass and volume [10]. A subset of CMR cases from the MESA baseline examination, together with contours from the CMR core laboratory and clinical information from the coordinating center, were contributed to the Cardiac Atlas Project (CAP) to provide a resource for cardiac image data sharing and atlas-based shape analysis for population studies [11].

Principal component analysis (PCA) is an established mathematical technique for quantifying the statistical variation of global shape in population atlases, and promises to provide novel information on the pathogenesis of neurological and cardiovascular disease [11]-[15]. However, the application of PCA in sub-clinical volunteers has not been investigated.

In this study, we sought to establish the most important components of LV shape and function (known as principal components) present in the MESA cohort contributed to CAP. Automated methods were developed to build an atlas from contour and landmark information. We hypothesized that the principal components of global LV shape in the asymptomatic population are associated with traditional indices of adverse remodeling, and that PCA can more clearly distinguish differences between sub-cohorts than current indices of remodeling such as mass and volume.

## Methods

### Participants

A total of 1,991 MESA CMR cases with matching contours and landmark information were obtained from the CAP database (www.cardiacatlas.org). These represented a random sample of the MESA baseline CMR examinations contributed to CAP with local Institutional Review Board approval. Informed participant consent compatible with sharing of de-identified data was obtained in all cases. Imaging studies and derived analyses were de-identified in a HIPAA compliant manner, annotated using standard ontological schema, stored in a web-accessible picture archiving and communication system (PACS) database, and analyzed using atlas-based techniques [11]. Participants were asymptomatic and free of clinical indications of cardiovascular disease at baseline, and standard risk factor profiles including smoking, hypertension, and fasting glucose data were obtained [9]. Ethnicity was determined by participant self-identification.

CMR was performed as part of the baseline MESA examination during 2000–2002. Images were acquired on 1.5 T scanners using a four element phased-array coil, as described previously [10]. Fast gradient recalled echo cine images were acquired with 10–12 short axis slices and one four chamber long axis slice (6 mm thickness, 4 mm gap, field of view 360–400 mm, 256 × 160 matrix, flip angle 20°, echo time 3–5 ms, repetition time 8–10 ms) with 20–30 frames per slice (temporal resolution <50 ms). The pixel size varied from 1.4 to 2.5 mm/pixel depending on patient size. Each slice was acquired in a separate breath-hold. Contours were manually drawn as a series of points by the MESA CMR core lab on short-axis slices for all cases at end-diastole (ED) and end-systole (ES) [10] using Q-MASS software (v. 4.2, Medis, the Netherlands). For a typical case with 7–10 short-axis slices, a total of ~2,000 contour points were available.

CMR LV mass and volume data for this cohort are shown in Table 1.

**Table 1:** CMR LV mass and volume calculated from the CMR core lab contours using slice summation

			**EDVI (ml/m**^**2.7**^**)**	**ESVI (ml/m**^**2.7**^**)**	**LVMI (g/m**^**2.7**^**)**	**EF (%)**
Total		1,991	31.7 ± 6.6	9.9 ± 3.9	36.7 ± 8.4	69 ± 8
Sex	Female	1,034	31.6 ± 6.1	**8.9 ± 2.9**	**35.1 ± 7.6**	**72 ± 6**
	Male	957	31.7 ± 7.1	**10.8 ± 4.5**	**38.4 ± 8.8**	**66 ± 8**
Ethnicity	White^1^	739	**30.6 ± 6.2**^**4**^	**9.7 ± 3.4**^**2,4**^	**35.2 ± 7.2**^**2,3,4**^	**67 ± 7**^**2**^
	Chinese^2^	356	**30.1 ± 5.0**^**3,4**^	**8.4 ± 2.5**^**1,3,4**^	**33.1 ± 6.3**^**1,3,4**^	**72 ± 6**^**1,3,4**^
	Black^3^	405	**31.8 ± 7.3**^**2,4**^	**10.4 ± 4.5**^**2**^	**38.4 ± 9.5**^**1,2**^	**68 ± 8**^**2**^
	Hispanic^4^	491	**34.4 ± 6.7**^**1,2,3**^	**10.8 ± 4.3**^**1,2**^	**39.9 ± 8.9**^**1,2**^	**69 ± 8**^**2**^
Smoking	Never^1^	1,053	31.8 ± 6.1	**9.5 ± 3.3**^**3**^	**35.9 ± 7.7**^**3**^	**70 ± 7**^**2,3**^
	Former^2^	682	31.5 ± 7.1	**10.1 ± 4.3**	**37.1 ± 9.0**	**68 ± 8**^**1**^
	Current^3^	249	32.0 ± 7.2	**10.7 ± 4.3**^**1**^	**38.3 ± 9.0**^**1**^	**67 ± 7**^**1**^
Alcohol	Never^1^	490	31.3 ± 5.8	**9.0 ± 3.1**^**2,3**^	**35.4 ± 7.7**^**2**^	**72 ± 7**^**2,3**^
	Former^2^	492	31.8 ± 7.2	**10.0 ± 4.3**^**1**^	**37.5 ± 8.9**^**1**^	**69 ± 8**^**1**^
	Current^3^	990	31.9 ± 6.7	**10.2 ± 3.9**^**1**^	**36.8 ± 8.4**	**68 ± 7**^**1**^
Hypertension	No	1,135	**31.2 ± 6.3**	9.9 ± 3.6	**34.8 ± 7.2**	**69 ± 7**
	Yes	856	**32.3 ± 7.0**	9.8 ± 4.2	**39.1 ± 9.2**	**70 ± 8**
Diabetes	Normal^1^	1,444	31.6 ± 6.5	9.8 ± 3.6	**35.8 ± 7.9**^**2,3,4**^	69 ± 7
	Impaired fasting glucose^2^	285	31.6 ± 6.3	9.7 ± 3.7	**37.8 ± 8.3**^**1**^	70 ± 8
	Untreated diabetes^3^	58	33.0 ± 6.8	11.0 ± 4.0	**40.9 ± 10.9**^**1**^	67 ± 9
	Treated diabetes^4^	203	32.5 ± 7.7	10.3 ± 5.4	**40.2 ± 9.4**^**1**^	69 ± 9

Significant differences by ANOVA (p < 0.005) are highlighted in bold-face. Scheffé’s multiple-comparison post-hoc tests are represented by super-indices indicating differences between the labeled sub-cohorts at an α-level of 0.005 (when significant differences were found in categorical variables).

### Atlas construction

**Figure 1:**
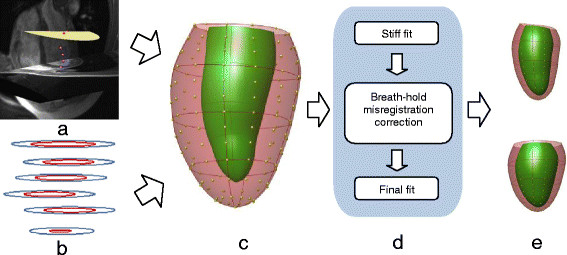
**Flow chart of the atlas construction. (a)** Fiducial landmarks defined at ED on short and long axis images (3D view from anterior). Red markers denote the mitral valve and purple markers denote the intersections of the right ventricular free wall and the septum. The base plane is drawn as a yellow disc. **(b)** Contours drawn on short axis slices by the core lab. Individual breath-holds for each 2D slice result in mis-alignment between slices. **(c)** 3D finite element model showing epicardial control points (model shape parameters) and element boundaries. **(d)** Breath-hold mis-registration correction by alignment to the model. **(e)** Principal component analysis of atlas shape variation. Upper and lower panels show ±2 standard deviations in the principal component shape.

The overall construction process for the atlas is shown in Figure 1. Fiducial landmarks were manually placed at the centroid of the LV cavity on the apical and basal ED short axis images, the hinge points of the mitral valve in the ED long axis image (Figure 1a), and at the insertions of the right ventricular free wall into the inter-ventricular septum in the ED short axis images. These were used to define a patient specific coordinate system which although individualized, was generally aligned in the same way for all patients, and initialize the position of the model, as described in the Appendix.

The model coordinates were used to provide the atlas coordinates of the LV: each point was assumed to be in the same anatomical location and this allowed alignment of the hearts of all patients [16].

### Automated model customization

Models were customized to each case by fitting the mathematical endocardial and epicardial surface model to the landmarks and short axis contours [17]. A 3D plane was fitted to the mitral valve hinge points to accurately represent the upper bound of the cavity as the mitral valve annulus. Mis-registrations of the contours due to changes in the breath-hold position from slice to slice (Figure 1b) were automatically corrected by shifting the contours in-plane to match an initial model fit (see Appendix).

### Validation

In order to validate the automated customization and breath-hold correction method, a random sample of 244 cases were independently analyzed by expert users who manually customized the models to each case using guide-point modeling blinded to the automated results [18]. The model was interactively customized to each case by placing guide points on the epicardial and endocardial images using custom software (CIM v. 6.0, Auckland, New Zealand). In-plane shifting of the slices was manually performed by the user to correct for breath-hold mis-registrations. This method has been previously validated against autopsy LV mass in animals, in patients against manually drawn contours, and in healthy volunteers against flow-derived measurements of cardiac output [18].

To calculate the error between the automated and manual methods, the sampled points from both manually customized and automatically generated shape models were pair-wise aligned by rigid body rotation to ensure maximum alignment using the Kabsch algorithm [19]. The error was then calculated as the root mean squared distance between corresponding points on both models.

### Atlas based analysis

**Figure 2:**
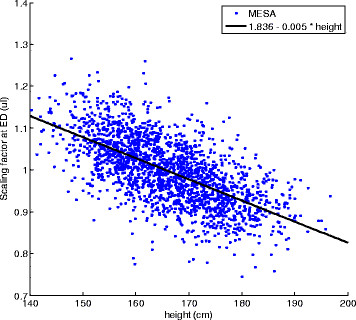
**Scale factor calculated from Procrustes alignment, plotted against body height.** The linear regression line (black) was used to provide a scale factor for each case to correct heart size for body habitus.

In atlas-based analyses, differences due to pose (translation and rotation) and scale (uniform size) are typically removed from all cases before performing the shape analysis. We used a scaled Procrustes alignment [20] to determine the pose and a scale factor for each shape model relative to the mean, and removed these effects. However, changes in heart size beyond that predicted by body habitus are known to occur due to disease processes. Removal of all size information would therefore be counterproductive in the current application. Figure 2 shows that the relationship between scale factor and height was approximately linear across the entire cohort. We therefore scaled each heart according to its height predicted scale factor obtained from the linear regression line, thereby retaining any residual size (i.e. not predicted by height) in the subsequent shape analysis.

Principal component analysis (PCA) was used to find the most important global shape variations in the atlas [11],[14]. Firstly, the three-dimensional positions of a large number of points uniformly placed on the smoothed epicardial and endocardial surfaces of the LV were calculated (Appendix). PCA finds the smallest number of statistically independent shapes, (or components), which explain as much of the global shape variation as possible [21] (see Appendix for details). Traditional measures of shape, such as volume, thickness, and dimensional shape changes, are thus inherently included within the principal shape components. The first component has the largest possible variance, which accounts for as much of the variability in the data as possible, and each succeeding component in turn explains as much of the residual variability as possible. Two separate PCAs were performed, using ED shapes and ES shapes separately.

In order to test the hypothesis that principal components are better than standard remodeling indices of mass and volume in determining shape differences between sub-cohorts, we applied linear discriminant analysis (LDA) to characterize shape differences [22]. This method provided the optimum way of distinguishing between sub-cohorts for both PCA and standard measures.

### Statistics

The strength of each component present in a particular case was calculated by projection (see Appendix) and normalized as a z score (mean of 0, standard deviation of 1.0 across the whole cohort). Comparisons of z scores for the first two components were tested using ANOVA for each dependent variable (sex, ethnicity, smoking, alcohol, hypertension and diabetes). Note that z-scoring has no effect in the ANOVAs. Post-hoc tests were performed using the Scheffé test. A more stringent p value of 0.005 was considered significant [23]. Linear discriminant analysis was applied to the first two components, the first 50 components, and standard remodeling measures of mass and volume. Effect size between sub-cohorts was quantified with Cohen’s d, which measures the mean difference in multiples of the pooled standard deviation.

## Results

### Validation

The error, quantified in 244 cases between the automatic and expert-derived models, was (mean ± std.dev.) 1.1 ± 0.6 mm at ED and 0.9 ± 0.5 mm at ES. The model fit error between the contour points and the model surfaces from the 1,991 cases was 0.5 ± 0.4 mm at ED and 0.5 ± 0.6 mm at ES. These were less than the pixel size of 1.4-2.5 mm.

### Atlas components

**Figure 3:**
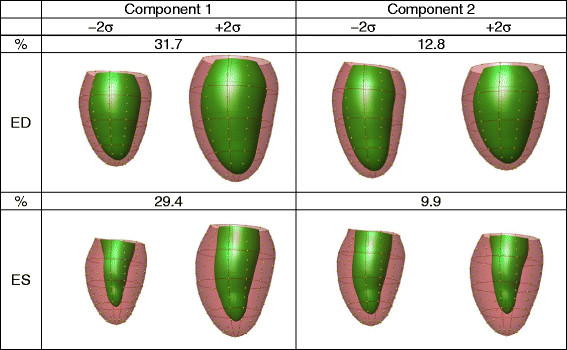
**First and second principal shape components of variation in the atlas (N = 1,991) for ED and ES.** For each component the left and right shapes represent the mean ± 2 std. dev. in the component distribution. Viewpoint is from the septum, posterior wall on the right.

Figure 3 shows the first and second principal components for each of the ED and ES atlases. At ED, the first principal component (component 1) accounted for 32% of the total variation (Figure 3 left, top) and visually, this component was primarily associated with heart size. We therefore call this component “size” for convenience although it is not a pure scaling. The second principal component at ED accounted for 13% of the total variation (Figure 3 right, top). Since this was associated with the height to width ratio we call this component “sphericity”, although it is not a pure height to width scaling.

At ES, 30% of the total variation was explained by the first principal component (Figure 3 left, bottom), which was associated with size (similar to ED component 1). The second component (10% of total variation) was associated with the ratio of cavity volume to wall volume (Figure 3 right, bottom). We call this component “concentricity” since higher amounts of this component lead to more concentric shape at ES. The third component (9%, not shown) was associated with ventricular sphericity at ES.

The strength of each component present was quantified (Appendix) and the z-scored weights of these coefficients in the first two principal components are shown in Table 2.

**Table 2:** Projection onto first two principal shape components for sub-cohorts

		**N**	**ED**		**ES**	
			**Component 1**	**Component 2**	**Component 1**	**Component 2**
			**“Residual size”**	**“Sphericity”**	**“Residual size”**	**“Concentricity”**
Sex	Female	1,034	**−0.16 ± 0.92**	**0.11 ± 0.98**	**−0.21 ± 0.88**	**0.52 ± 0.82**
	Male	957	**0.17 ± 1.05**	**−0.11 ± 1.01**	**0.22 ± 1.07**	**−0.56 ± 0.86**
Ethnicity	White^1^	739	**−0.18 ± 0.94**^**3,4**^	−0.04 ± 0.97	**−0.12 ± 0.90**^**2,3,4**^	**−0.30 ± 0.96**^**2,4**^
	Chinese^2^	356	**−0.34 ± 0.80**^**3,4**^	−0.05 ± 0.90	**−0.49 ± 0.77**^**1,3,4**^	**0.30 ± 0.84**^**1,3**^
	Black^3^	405	**0.11 ± 1.08**^**1,2,4**^	−0.02 ± 1.08	**0.24 ± 1.12**^**1,2**^	**−0.22 ± 1.01**^**2,4**^
	Hispanic^4^	491	**0.42 ± 1.00**^**1,2,3**^	0.11 ± 1.03	**0.34 ± 1.01**^**1,2**^	**0.42 ± 0.96**^**1,3**^
Smoking	Never^1^	1,053	−0.04 ± 0.94	0.04 ± 0.97	**−0.08 ± 0.93**^**3**^	**0.18 ± 0.99**^**2,3**^
	Former^2^	682	0.03 ± 1.06	−0.05 ± 1.02	**0.03 ± 1.07**	**−0.19 ± 0.98**^**1**^
	Current^3^	249	0.10 ± 1.08	−0.04 ± 1.04	**0.24 ± 1.06**^**1**^	**−0.24 ± 0.97**^**1**^
Alcohol	Never^1^	490	**−0.14 ± 0.89**^**3**^	0.05 ± 0.94	**−0.21 ± 0.90**^**2,3**^	**0.46 ± 0.89**^**2,3**^
	Former^2^	492	**0.02 ± 1.07**	0.02 ± 1.10	**0.05 ± 1.06**^**1**^	**0.01 ± 0.97**^**1,3**^
	Current^3^	990	**0.06 ± 1.01**^**1**^	−0.03 ± 0.98	**0.08 ± 1.01**^**1**^	**−0.23 ± 0.98**^**1,2**^
Hypertension	No	1,135	**−0.09 ± 0.97**	**−0.10 ± 0.94**	**−0.08 ± 0.93**	**−0.16 ± 0.97**
	Yes	856	**0.12 ± 1.03**	**0.14 ± 1.06**	**0.10 ± 1.07**	**0.21 ± 1.01**
Diabetes	Normal^1^	1,444	−0.04 ± 1.00	−0.03 ± 1.00	−0.04 ± 0.98	**−0.07 ± 0.99**^**4**^
	Impaired fasting glucose^2^	285	0.06 ± 0.94	0.01 ± 0.93	0.05 ± 0.99	**0.15 ± 0.95**
	Untreated diabetes^3^	58	0.23 ± 1.05	0.23 ± 1.08	0.25 ± 1.08	**0.22 ± 1.12**
	Treated diabetes^4^	203	0.16 ± 1.08	0.11 ± 1.05	0.16 ± 1.13	**0.24 ± 1.05**^**1**^

Z-scores of the projected weights are shown for each sub-cohort and component (mean ± std. dev.). Significant differences (p < 0.005) are highlighted in bold-face. Scheffé’s multiple-comparison post-hoc tests are represented by super-indices indicating differences between the labeled sub-cohorts at an α-level of 0.005 (when significant differences were found in categorical variables). Sub-cohorts may not add to 1991 due to missing data.

Table 2 shows that significant differences were found due to sex at ED, with males having larger size (z score 0.17 vs −0.16, or a difference of 33% of the cohort standard deviation), and less spherical hearts (22%). At ES male hearts were larger (43%) and less concentric (108%). For ethnicity, significant differences in residual size were found at ED, with Hispanics having the largest hearts, and Chinese the smallest, with a difference of 76% between these groups (after correction for height). This pattern was preserved at ES, but Hispanics were also the most concentric (72% difference from Whites).

Among risk factor sub-cohorts in Table 2, smoking was associated with greater size and less concentricity at ES, with increasing effect from Never to Former to Current (32% for size and 44% for concentricity between Never and Current). Similarly alcohol consumption was associated with greater size at ED (20%) and ES (29%) and less concentricity at ES (69%), between Never and Current. Hypertension was associated with larger size (21%) and sphericity (24%) at ED and larger size (18%) and more concentricity (37%) at ES. Diabetes was associated with higher concentricity at ES, with increasing effect from Normal to Impaired Fasting Glucose to Untreated Diabetes to Treated Diabetes (31% between Normal to Treated).

Table 3 shows the results of the LDA for the first two components, the first 50 components, and standard indices. The two component LDA showed more significant differences (lower p value) and greater effect sizes (measured with Cohen’s *d*[24]) than standard measures in most cases. The 50 component LDA showed greater discriminatory power than both.

**Table 3:** Linear discriminant analysis for first two principal shape components (LDA2) and first 50 components (LDA50) compared with standard remodeling indices (Standard)

		**LDA2**		**LDA50**		**Standard**			
		**ED**	**ES**	**ED**	**ES**	**EF**	**EDVI**	**ESVI**	**LVMI**
Smoking	-log(p)	2	21	46	46	11	0	5	4
	Cohen’s *d*	0.13	0.43	**0.67**	0.66	**0.30**	0.02	0.20	0.18
Diabetes	-log(p)	4	9	49	48	0	1	1	14
	Cohen’s *d*	0.18	0.30	**0.77**	0.75	0.02	0.08	0.07	**0.40**
Hypertension	-log(p)	12	20	113	101	5	3	0	30
	Cohen’s *d*	0.33	0.42	**1.09**	1.03	0.20	0.16	0.03	**0.53**
Sex	-log(p)	19	182	>200	>200	62	0	28	18
	Cohen’s *d*	0.41	1.44	**2.34**	2.23	**0.78**	0.02	0.51	0.40
White	-log(p)	9	29	93	96	2	8	1	8
	Cohen’s *d*	0.29	0.53	1.01	**1.02**	0.12	**0.27**	0.08	0.27
Chinese	-log(p)	12	33	79	98	16	7	15	19
	Cohen’s *d*	0.43	0.72	1.16	**1.30**	0.49	0.30	0.48	**0.53**
Black	-log(p)	2	13	81	67	5	0	3	6
	Cohen’s *d*	0.15	0.41	**1.12**	1.01	0.25	0.02	0.18	**0.27**
Hispanic	-log(p)	28	44	93	79	0	26	9	23
	Cohen’s *d*	0.59	0.75	**1.13**	1.03	0.02	**0.57**	0.32	0.53

For ethnicity each test compares one group with the rest (e.g. white vs. non-white). Significance is quantified by –log(p) (e.g. for p = 0.001, −log(p) = 3). Effect size is measured by Cohen’s d, which can be interpreted as the mean distance between two groups in standard deviations (e.g. males and females were separated by 2.34 standard deviations in the LDA50 ED analysis). Bold-faced numbers highlight the highest separation achieved by PCA vs. standard remodeling indices.

## Discussion

Although atlas-based shape analysis is well established in neurological imaging [12],[13], application to cardiac disease has been limited [11],[14],[15]. Atlas-based analysis has the potential to reveal new measures of geometry and function, which may provide novel insights into the remodeling processes of disease. For example, Lewandowski *et al.*[15] recently showed significant differences in principal components between individuals born preterm (30 weeks) and full-term age matched healthy volunteers, which are indicative of cardiac remodeling associated with the premature switch to post-natal circulation.

There has been limited information on the distribution of global shape inherent in the asymptomatic population. We have created one of the largest atlases of the left ventricle to date, built from 1,991 models. The data and results are available on request from www.cardiacatlas.org. The main application of this atlas is to quantify adverse remodelling in clinical patients, standardized against the asymptomatic population atlas, by calculating the strength of each principal component present in a particular patient. Changes over time, or the effect of treatment, can then be precisely quantified by progression of the component coefficients.

Our results showed that the first principal component of shape variation at ED and ES was associated with heart size, accounting for about 30% of the total variance. Significant differences were found in this component due to sex, ethnicity, smoking (ES only), alcohol, and hypertension. This confirms the importance of heart size as the dominant morphological index, even after correction for body size. The differences in the size component between sub-cohorts (Table 2) mirrored the differences in mass and volume (Table 1). In asymptomatic individuals, heart size has been associated with adverse events in the Framingham study using chamber dimension [6], and with heart failure in the MESA cohort using EDV and LV mass [25]. Volume and mass also differed with standard risk factors of smoking, hypertension and diabetes in the MESA cohort [26]. In patients with myocardial infarction, ESV is a strong predictor of mortality [2]. ESV also predicted adverse events in high risk patients in the ONTARGET study [27].

The second component of shape variation was associated with sphericity at ED, and this was also associated with the third component at ES. In a CMR study of 120 asymptomatic volunteers, women had more spherical ventricles than men [28], in agreement with our results. In patients with myocardial infarction, sphericity has been associated with decreased survival [3] and is a predictor of future gain in LV volume [4]. Sphericity was also shown to be the primary geometric determinant of functional mitral regurgitation in heart failure [29]. Cavity shape was more spherical in hypertensive patients with eccentric hypertrophy [30]. In advanced idiopathic dilated cardiomyopathy, increased sphericity has been linked to poorer survival [6], increased metalloproteinase activity and loss of collagen [31]. Our results support the hypothesis that sphericity is an important shape descriptor in asymptomatic individuals as well as patients. Atlas-based sphericity measures can be simply calculated in individuals by projection onto the sphericity component. Since the components are orthogonal, this measure of sphericity is independent from the other components of shape variation.

The second component at ES was associated with the ratio of wall thickness to cavity size, which is indicative of concentric remodeling [30]. Further work is needed to determine if this component was due primarily to wall thickening, i.e. a difference between ED and ES, or with wall thickness at ED. However, this component of remodeling is well known to be clinically associated with adverse outcomes [5]. In the Framingham study of asymptomatic volunteers, concentric remodeling was associated with increased risk of incident cardiovascular disease [32].

It is important to note that the shapes produced by PCA are determined solely by the data, and each contains global shape information pertaining to all regions. The use of the terms ‘size’ and ‘sphericity’ are ways of labelling the components in a clinically meaningful way, but the shape information is more complex than this as it varies regionally in a complex way as determined by the data.

### Limitations

Limitations of this study include the requirement for manual contouring and identification of landmarks in the construction of the atlas. However, methods are becoming available for the fully automated determination of LV contours and fiducial landmarks [33],[34]. In the near future it will be possible to generate large scale cardiac atlases fully automatically. Another limitation is that all CMR studies in the baseline MESA examination used the fast gradient recalled echo protocol. The current standard for cine CMR is steady state free precession and this was used for the recent ten year follow-up examination in MESA. It is well known that these protocols give rise to different mass and volume measurements, and also result in shape bias [35]. Methods to remove such bias from models for the purposes of shape comparison show promise [35]. These methods can be applied in future studies to examine the shape changes between baseline and follow-up MESA examinations. Future work is also required to determine whether the strength of the primary components is related to outcomes.

Due to the contouring and model fitting process, individual variations of trabecular prominence and relatively small features such as ‘crypts’ are not captured in the global shape analysis. However, measures of trabeculation (such as those described in [36]) can be correlated with our dimensional shape measures, which could identify possible relationships between shape and trabeculation.

Mitral annular dimensions are captured in the shape model, since the model surfaces are defined up to the mitral valve, which is considered as a simple plane. Mitral valve plane variations were seen in the principal components but have less power than size, sphericity and concentricity. However, partial voluming in the short axis images near the mitral valve contributes to variability in the measurement. More detailed modelling of the mitral annulus shape would provide more insight into shape changes [37].

Other shape features of clinical interest, such as LV wall thickness and thickening, are also present in the principal components. However they are not as powerful as other components since the MESA participants were asymptomatic and were not recruited for a particular disease phenotype. If a PCA were constructed using a significant number of HCM or DCM patients, we would expect that the components would directly reflect wall thickening or thinning in these patients. The use of quasi-orthogonal components defined with a clinical rationale, such as hypertrophic or dilated cardiomyopathies, is an interesting area of future research.

## Conclusions

In conclusion, we have established the range of dimensional shape variation in adult asymptomatic individuals. Shape was dominated by components associated with size, sphericity, and concentricity. These components have been associated in the literature with adverse remodeling, and future work is needed to determine if these components are also associated with adverse outcomes in asymptomatic volunteers.

## Competing interests

The authors declare that they have no competing interests.

## Authors’ contributions

PM-G drafted the manuscript and participated in the study design, data analysis, data interpretation and manuscript editing. BRC participated in the study design, data interpretation and manuscript editing. BA-V participated in the data interpretation and manuscript editing. DAB participated in the study design, data acquisition and manuscript editing. JE participated in the study design, data interpretation and manuscript editing. JPF participated in the study design, data interpretation and manuscript editing. CGF participated in the study design, data interpretation and manuscript editing. JACL participated in the study concepts, data acquisition and manuscript editing. AS participated in the study design, data analysis and manuscript editing. AAY conceived the study and participated in the study design, data interpretation and manuscript editing. All authors read and approved the final manuscript.

## Appendix

The model comprised 16 bicubic finite elements defined in a prolate spheroidal coordinate system. This enabled an efficient representation of the shape of the left ventricle as a radial function of two angular coordinates: λ(μ,θ) (see [18],[38] for details). Shape parameters of the model were obtained from the epicardial and endocardial surfaces. These parameters were evenly spaced around the heart and intuitively control the position of the model locally at each point. Figure 1c shows the spatial distribution of the shape parameters.

The model was initialized by aligning and scaling the generic model to the patient-specific coordinate system and fiducial landmarks of each case. The model shape parameters were then automatically fitted to the contour points by linear least squares constrained optimization, which minimized the surface error between the contour points and the corresponding model points:

(1) $$E\left(\lambda \right)=\mathrm{wS}\left(\lambda \right)+\sum_{c}{\left(\lambda -\lambda_{c}\right)}^{2}$$

subject to the constraint that the epicardial surface had to be placed outside the endocardial surface. Eqn. 1 was minimized as a quadratic programming problem using MATLAB’s Optimisation Toolbox (v. 6.0, The MathWorks Inc., Natick, MA, USA). The influence of the smoothing term S(λ) was controlled though the size of the weight *w*: higher *w* gives less ripples in the surface (for details see [39]). Controlling S(λ) is important since there is a trade-off between smoothing and resolution, i.e. a highly smooth model might better represent the anatomy from a global point of view, but might blur out smaller-scale anatomical details.

**Figure 4:**
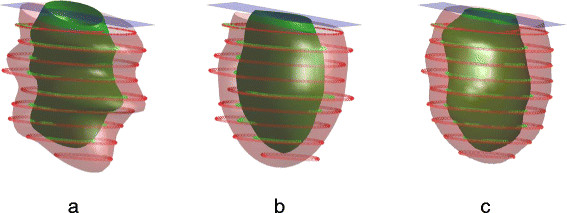
**Breath-hold correction. (a)** original contours with a superimposed low- stiffness fit; **(b)** highly stiff model which serves as a guide for breath-hold correction; **(c)** final fit with low stiffness and corrected contours. Epicardial contours and surfaces are shown in blue; endocardial contours and surfaces are shown in red.

Due to differences in breath-hold position between slices, the model can exhibit artifactual ripples in the longitudinal direction (Figure 4a). Although breath-hold mis-registrations can result in 3D rotations and translations for each slice, to a first order approximation these can be substantially removed by in-plane translations of the short axis images [40]. To correct these mis-registrations, a three-step process was employed. Firstly, Eqn. 1 was minimized using high stiffness weights (*w* = 100) to fit the manual uncorrected contours (Figure 4b). This provided a case-specific smooth version of the model without longitudinal ripples. Secondly, the intersection of the image plane with the stiff model generated a new contour. An in-plane shift vector was defined between the centroids of the manual contour and the stiff model contour, and the manual contour was shifted so that its centroid coincided with the centroid of the stiff model contour. This ensured that the shifts aligned the contours to a smooth version of the anatomy. A final fit with low smoothing weight (*w* = 1) was then performed to obtain the customized geometry (Figure 4c).

In order to calculate statistical shape differences, the endocardial and epicardial surfaces were sampled at high density using a standardized sampling pattern (10 × 10 points per element) and the positions converted to rectangular Cartesian coordinates in mm. This led to a total of 2738 points per model (duplicate points on element boundaries were removed). This represented an oversampling of the model shape in order to ensure that all shape characteristics were included to the extent of the contour resolution.

Principal component analysis (PCA) was applied to the sampled points from all models [11],[14]. Briefly, the sampled surfaces were aligned to remove variation due to translation, rotation, and scale due to patient height. The aligned point positions were assembled into a column vector for each case. The mean position of each point was calculated by the arithmetic average across cases and subtracted. Vectors from all cases were then assembled into a shape matrix *B*. The covariance matrix was calculated as

(2) $$C\;=\;N^{-1}BB^{T}$$

where *N* is the number of cases (*N =* 1,991).

In PCA, eigenvectors of the covariance matrix are computed to give the principal components present in the multi-variate point distributions, and their corresponding eigenvalues give the *variance* for each component [21]. Typically, most of the variation can be explained by relatively few components due to redundancy in the shape vectors. The first principal component shape is associated with the largest eigenvalue and explains the greatest variance in the data. Each subsequent principal component explains the maximum residual variation possible. The strength of the principal component present in a particular case can be found by projection of the shape vector onto the *direction* defined by the corresponding eigenvector.
